# Supplementary material for: Recruiting and Engaging Women of Reproductive Age with Obesity: Insights from A Mixed-Methods Study within A Trial
Source: Int J Environ Res Public Health. 2022 Oct 24;19(21):13832. doi: 10.3390/ijerph192113832 (PMC9658053; doi:10.3390/ijerph192113832)
Supplement: Supplementary file 1 [file ijerph-19-13832-s001.zip › ijerph-1945828-supplementary.pdf]

Table S1: Questions from the GetGutsy SWAT electronic survey

Question	Responses
Before the GetGutsy Study, had you interest in taking part in a research study before?	Yes or no<br>If yes, select the stage of the research study you had completed. Choose all that apply<br>• Applied to take part<br>• Started study<br>• Completed study
How did you hear about the GetGutsy study? Please select all that apply	• Heard by email<br>• Heard by ezine/newsletter<br>• Phone text social media<br>• Recruitment stand<br>• Told by friend or relative<br>• Told by colleague<br>• Poster at work<br>• Poster outside work<br>• Radio<br>• Browsing social media<br>• Other
What are the 3 ways you would like to find out about research studies?	Choose three from:<br>• By direct email<br>• By ezine or e-newsletter<br>• By social media (such as twitter, Facebook, Instagram)<br>• By speaking to the researchers at a recruitment stand (at work or university<br>• By speaking to the researchers at a recruitment stand (in a public place)<br>• By reading a poster or leaflet on a notice board<br>• By hearing about it from a friend or relative<br>• By hearing about it from a work colleague<br>• Other
What motivated you to contact the research team about the study?	Choose one from:<br>• I was interested in the health screen<br>• I was interested in getting the probiotic<br>• I wanted to support health research<br>• I wanted to be more healthy<br>• All the above

Out of the following options, which 3 interest you the most?	Choose three from:<br>• Free blood test<br>• Feedback on my dietary habits<br>• Feedback on my lifestyle habits<br>• Knowledge of my height, weight & body composition<br>• Trying a new probiotic intervention<br>• Helping to support health research<br>• Other
At what point did you contact the researchers after first hearing about the study?	Choose one from:<br>• Same day<br>• A day or two later<br>• One week later<br>• Two weeks later<br>• Greater than two weeks later
A weight criterion would:	Likert scale for each option (Strongly disagree to strongly agree)<br>• Stop me from sharing this study with work colleagues<br>• Stop me from sharing this study with my friends or family<br>• Makes me less likely to contact the researchers about taking part
Would the following options make you more or less likely to take part in a research study?	Likert scale for each option (Strongly disagree to strongly agree)<br>• Having a 50% chance of receiving a placebo instead of a probiotic<br>• Required to give a blood sample<br>• Required to give a stool sample<br>• Travelling to UCD for a study appointment<br>• Travelling to the National Maternity Hospital for a study appointment<br>• Attending a study appointment Monday to Friday (during work hours)<br>• My work colleagues knowing I am taking part in the study

Demographic questions are not included

Table S2: Semi-structured topic guide for GetGutsy SWAT Focus Groups

Main questions	Prompts / related questions
How did you get involved with the GetGusty study?	- How did you find out about the study?<br>- How did you get in contact with the researchers?
What was it like to take part in the GetGutsy study?	- How was it communicating with the researchers / attending study visits?<br>- What interested you about the study offering?<br>- How did it compare to expectations?
What was the motivation behind wanting to take part?	- Were there any benefits to taking part?
What do you think about the things you got measured as part of the study?	- What did you think of the information you received / measures included?<br>- Was there anything missing? What would you measure in the future?<br>- How do you feel about including body mass index as an inclusion criterion in the GetGutsy study?<br>- What do you think about weight as a marker of health?<br>- How did this affect your comfort with sharing the study with others?<br>- How can we best measure health?<br>- How to define health and a healthy person?<br>- What aspects of health are most important?
